# Association between Paleolithic diet fraction and systemic low-grade chronic inflammation in the Malmö diet and cancer study cohort

**DOI:** 10.1007/s00394-025-03838-z

**Published:** 2025-11-12

**Authors:** Pedro Carrera-Bastos, Björn Rydhög, Yvonne Granfeldt, Kristina Sundquist, Emily Sonestedt, Peter M. Nilsson, Tommy Jönsson

**Affiliations:** 1 Center for Primary Health Care Research, Department of Clinical Sciences in Malmö, Lund University, Malmö, Sweden; 2 University Clinic Primary Care, Skåne University Hospital, Region Skåne, Malmö, Sweden; 3 Department of Process and Life Science Engineering, Lund University, Lund, Sweden; 4 Nutritional Epidemiology, Department of Clinical Sciences in Malmö, Lund University, Malmö, Sweden; 5 Department of Food and Meal Science, Faculty of Natural Science, Kristianstad University, Kristianstad, Sweden; 6 Internal Medicine – Epidemiology, Department of Clinical Sciences in Malmö, Lund University, Malmö, Sweden

**Keywords:** C-reactive protein, Malmö diet and cancer study, Neutrophil-to-lymphocyte ratio, Paleolithic diet fraction, Systemic low-grade chronic inflammation, Total leukocyte count

## Abstract

**Purpose:**

The Paleolithic Diet Fraction (PDF) estimates the proportion of absolute dietary intake derived from food groups included in the Paleolithic diet. In the Malmö Diet and Cancer Study (MDCS), higher PDF and lower systemic low-grade chronic inflammation (SLGCI) have been associated with lower cardiometabolic morbidity and mortality. We examined associations between PDF and SLGCI in the MDCS.

**Methods:**

The study population (*n* = 23,250; 63% women; ages 44–74 years) excluded participants with prior coronary events, diabetes, stroke, high-grade inflammation, or missing baseline covariate data. PDF was calculated from baseline dietary data collected via food frequency questionnaires, seven-day food records, and interviews. Biomarkers of SLGCI included total leukocyte count (TLC) and neutrophil-to-lymphocyte ratio (NLR) measured at baseline, and C-reactive protein (CRP) measured ~ 4 months later in a subpopulation (*n* = 4196).

**Results:**

PDF was significantly and inversely associated with all three biomarkers of SLGCI in both simple and fully adjusted models (adjusted for age, sex, physical activity level, BMI, smoking status, education level, living alone, born in Sweden, season of dietary data collection, and dietary method version): TLC (*B* = −0.008), NLR (*B* = −0.003), and lnCRP (*B* =  −0.005), respectively (*p* < 0.001). Inflammatory biomarkers were weakly but significantly correlated: TLC with NLR (*r*_*s*_ = 0.263), TLC with CRP (*r*_*s*_ = 0.262), and NLR with CRP (*r*_*s*_ = 0.062) (*p* < 0.001).

**Conclusion:**

PDF was inversely associated with SLGCI biomarkers, suggesting that SLGCI may mediate its relationship with cardiometabolic outcomes. Given the cross-sectional design and CRP measurement lag, these findings should be interpreted with caution.

**Supplementary Information:**

The online version contains supplementary material available at 10.1007/s00394-025-03838-z.

## Introduction

Systemic low-grade chronic inflammation (SLGCI) is characterized by a subtle yet persistent activation of various immune cells [1]. SLGCI may contribute to the onset and progression of chronic non-communicable conditions, including metabolic syndrome, type 2 diabetes, and cardiovascular disease [1–3], which are leading causes of morbidity and mortality worldwide [4, 5].

The assessment of SLGCI commonly relies on blood-based biomarkers [1, 6, 7], with each providing distinct insights into inflammatory status. Total leukocyte count is a widely used biomarker that reflects overall immune activation and has been positively associated with the risk of cardiovascular and metabolic diseases [2, 8–11]. The neutrophil-to-lymphocyte ratio (NLR) represents the balance between innate (neutrophils) and adaptive immunity (lymphocytes) [12]. NLR has been positively associated with the risk of metabolic syndrome [13], type 2 diabetes [14], and cardiovascular disease [15], in addition to all-cause and cardiovascular mortality [16, 17]. C-reactive protein (CRP), an acute-phase protein indicative of systemic inflammation [7, 18], has also been positively associated with the risk of type 2 diabetes [19], cardiovascular disease [7], and both cardiovascular and all-cause mortality [20, 21].

SLGCI can be modulated by diet, as demonstrated by multiple lines of evidence, including animal experiments, observational studies, and randomized controlled trials (RCTs) [22–30]. For example, cross-sectional studies have shown that diets rich in fruits, vegetables, nuts, seeds, and fish are associated with lower circulating levels of SLGCI biomarkers [26–30]. Concordantly, RCTs have found that several healthy dietary patterns, some of which specifically emphasize these foods, can significantly decrease biomarkers of SLGCI [24–26, 31]. Among these dietary patterns, the Paleolithic diet has, in a few RCTs, resulted in lower CRP levels compared to control diets [31].

The Paleolithic diet is modeled after the presumed dietary habits of pre-agricultural humans and emphasizes the intake of fruits, vegetables, roots, tubers, lean meats, fish, eggs, nuts, and seeds, while excluding grains, dairy, legumes, ultra-processed foods, as well as added sugars and fats [32, 33]. Observational studies using dietary scoring methods have found that dietary patterns resembling the Paleolithic diet are inversely associated with cardiometabolic disease incidence and both cardiovascular and all-cause mortality [34, 35]. In interventional studies, the Paleolithic diet has also resulted in beneficial effects on cardiometabolic health biomarkers, including lipid profiles, glucose regulation, and blood pressure [31, 36, 37].

To estimate how large a portion of the absolute dietary intake is derived from food groups included in the Paleolithic diet, the Paleolithic Diet Fraction (PDF) was developed [38]. PDF has in RCTs been found to be associated with better cardiometabolic health biomarkers [38, 39]. A higher PDF has also been associated with a reduced incidence of cardiometabolic disease and lower rates of cardiovascular and all-cause mortality in the Malmö Diet and Cancer Study (MDCS), Sweden [34]. MDCS was a large, population-based prospective cohort study that included 24,104 participants aged 44–74 years, 63% of whom were women [34]. Interestingly, in the MDCS, biomarkers of SLGCI were measured and found to also be associated with the incidence of cardiometabolic disease [2, 8]. Both PDF and SLGCI biomarkers have thus been separately associated with cardiometabolic morbidity in the MDCS. Our aim was to build upon these findings by examining associations between PDF and SLGCI biomarkers in the MDCS.

## Methods

### Study population

**Fig. 1:**
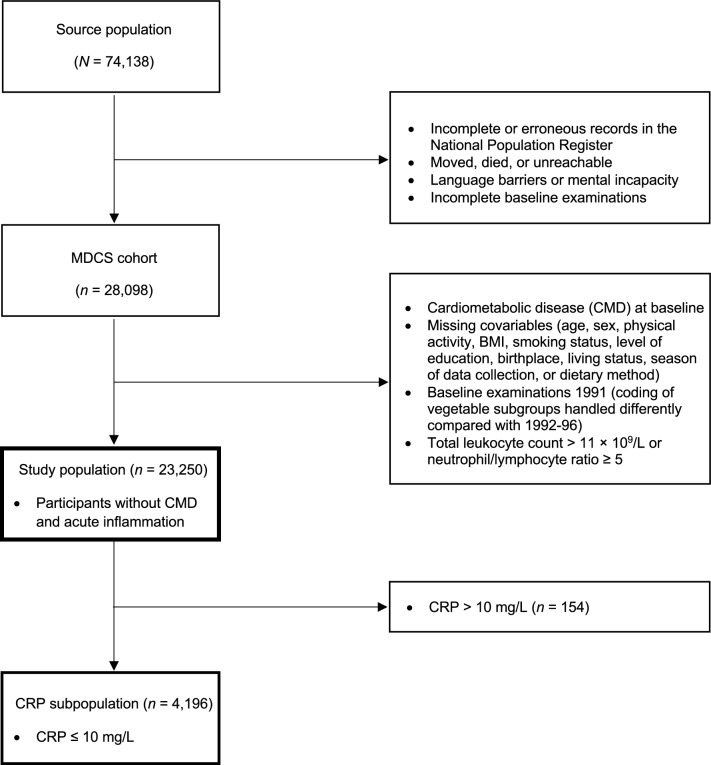
Study population flowchart. *Note*. Flowchart depicting participant selection from the Malmö Diet and Cancer Study (MDCS). The study population and CRP subpopulation were comprised of participants from the Malmö Diet and Cancer Study (MDCS) without previous coronary events, diabetes, stroke, or high-grade inflammation, and with no missing covariate data at baseline (1992–96). BMI, Body mass index; CMD, cardiometabolic disease; CRP, C-reactive protein. MDCS, Malmö Diet and Cancer Study

The MDCS is a population-based cohort study conducted in Malmö, southern Sweden. Baseline examinations occurred between 1991 and 1996, targeting all women born between 1923 and 1950 and all men born between 1923 and 1945 residing in Malmö (*N* = 74,138) [40]. Of these, a total of 28,098 participants completed all baseline examinations, which included dietary assessments, physical evaluations, and questionnaires addressing socioeconomic and lifestyle factors, as described in previous publications [34, 40]. For the present analysis, participants with type 2 diabetes, cardiovascular disease, or stroke at baseline were excluded, along with individuals missing covariate data necessary for regression analysis (age, sex, physical activity level, body mass index [BMI], smoking status, education level, living alone, born in Sweden, season of data collection, or dietary method). Participants who underwent baseline examinations in 1991 were also excluded because dietary data from that year did not support an accurate PDF calculation. Additionally, participants with a total leukocyte count above 11 × 10^9^/L or NLR above or equal to 5, indicative of acute high-grade inflammation [41, 42], were also excluded, resulting in a study population of 23,250 participants (see flowchart in Fig. 1). A sub-cohort of MDCS was randomly selected between 1991 and 1994 to study the epidemiology of carotid artery disease [43]. Among this sub-cohort, fasting plasma samples were collected approximately four months after the MDCS baseline examinations and analyzed for CRP, fasting blood glucose, hemoglobin A1c (HbA1c), serum insulin, and blood lipids. For the present study, a subpopulation of 4,196 participants was formed from participants of this sub-cohort with CRP values below 10 mg/L (values above 10 mg/L indicative of acute high-grade inflammation [7]) (Fig. 1).

All participants provided written informed consent, and the study was approved by the Regional Ethics Review Board in Lund, Sweden (Dnr. LU51-90).

### Dietary data and PDF

The dietary intake of participants in the MDCS was assessed at baseline using a modified diet history method designed to capture habitual dietary patterns. This method integrated three components: (1) a seven-day food record where participants logged meals with varying content, typically lunches and dinners, alongside records of cold beverages and supplements; (2) a 168-item food frequency questionnaire, estimating portion sizes and frequencies for commonly consumed foods with low day-to-day variation; and (3) a 45-min interview conducted by trained dieticians to validate records, clarify portion sizes using visual aids, and collect additional details on food preparation methods. This approach has been described in detail in prior MDCS publications [34, 44, 45].

Dietary data were processed using a specialized database and software incorporating standard recipes, food codes, and nutrient information. Nutrient values were sourced from the PC-KOST2-93 database of the Swedish National Food Administration and supplemented with cohort-specific recipes. The dietary data were grouped into aggregated categories to facilitate analysis, accounting for both nutrient content and dietary behavior relevance [45]. The reliability and validity of the dietary data were previously evaluated through comparisons with weighed food records, showing strong correlation coefficients for various food and nutrient intakes, thus supporting the robustness of the MDCS dietary assessment method [34].

To account for methodological differences, adjustments were made based on the time of dietary data collection. Specifically, data collected after September 1994 underwent revised coding routines that were introduced to streamline the dietary interview process. The adjustments included standardized coding for mixed dishes and modifications in portion-size estimation, changes which prior validation studies have shown to have minimal impact on ranking participants by dietary intake [45].

PDF was calculated as the fraction of total daily intake (by weight) from food groups consistent with the Paleolithic dietary model, excluding non-caloric beverages (e.g., water, coffee, and tea). Based on previously established criteria [34, 38, 39] and the MDCS food grouping system, food groups classified as Paleolithic were vegetables, fruits, potatoes, eggs, meat, fish, olive and rapeseed oil, nuts, and wine. Food groups classified as non-Paleolithic included legumes, juice, meat products (e.g., offal as mixed products or spreads, sausage), milk and milk products, sweet beverages, cereals (including rice), fats/oils and margarine, bakery sweets, jam, sauces and soups, beer, spirits, and miscellaneous items. In previous studies using detailed weighed food records, PDF could be calculated using the energy contribution of each food group [38, 39]. In the MDCS, however, the integrated dietary assessment method was not designed to yield precise, disaggregated energy estimates for individual food groups after merging all components (food record, questionnaire, and interview) [44, 45]. This limitation arises from the coding structure and aggregation procedures in the MDCS nutrient database, which link portion sizes and nutrient values to mixed dishes and aggregated food codes rather than to individual ingredients. Consequently, although total daily energy intake is available, it is not possible to obtain accurate energy values separately for Paleolithic and non-Paleolithic food groups. Therefore, PDF in the MDCS could only be computed on the basis of food weight, consistent with prior work in this cohort [34].

### Assessment of inflammatory biomarkers

Total leukocyte count and its subtypes (neutrophils and lymphocytes) were measured in heparinized blood samples at baseline using the SYSMEX K1000 automated hematology analyzer. NLR was calculated as the ratio of neutrophil count to lymphocyte count. CRP was measured in fasting plasma samples stored at −80 °C using the Tina-quant® CRP latex high-sensitivity assay (Roche Diagnostics).

### Other variables

Data on age and sex were obtained from national population registers using participants’ personal identification numbers. BMI was calculated from measured weight (kg) and height (m) and categorized into four groups: underweight (< 18.5 kg/m^2^), normal weight (18.5–24.99 kg/m^2^), overweight (25–29.99), and obesity (≥ 30 kg/m^2^) [46]. Leisure-time physical activity was assessed through self-reported weekly duration (minutes) of engagement in 17 different activities. Each activity was weighted by an intensity coefficient, and the resulting score was divided into sex-specific quintiles to account for differences in physical activity patterns [47]. Smoking status was classified into four groups: regular smokers (daily), occasional smokers (less than daily), former smokers, and never smokers. Education level was divided into four categories: ≤ 8 years of schooling, 9–10 years, 11–13 years, or university degree. Season of dietary data collection was categorized as winter, spring, summer, or fall (autumn) to account for seasonal variations. A binary variable labeled “dietary method version” was introduced to address methodological differences arising from slightly altered coding routines implemented in September 1994, as described above. Additional covariates included living alone (yes/no) and born in Sweden (yes/no).

### Statistical analysis

Associations between PDF and inflammatory biomarkers (total leukocyte count, NLR, and lnCRP) were assessed using simple and two multiple linear regression models. The first model was adjusted for age and sex. The second model was additionally adjusted for physical activity level, BMI, smoking status, education level, living alone, born in Sweden, season of dietary data collection, and dietary method version. Covariates were selected based on face validity and prior evidence of their potential to act as confounders, as documented in the literature and previous analyses of the MDCS cohort. In sensitivity analyses, associations between PDF and inflammatory biomarkers in fully adjusted multiple linear regression were also analyzed stratified by sex, age (tertiles), and PDF (tertiles), and the interaction between PDF and the stratified covariates was formally tested by adding corresponding interaction terms. Exploratory analyses of associations between food groups and inflammatory biomarkers were also performed using fully adjusted multiple linear regression. Correlations between variables were assessed using Pearson or Spearman coefficients, as appropriate. Statistical significance was set at *p* < 0.05. Analyses were performed in SPSS statistical software package (IBM, version 29.0.2.0).

## Results

Baseline clinical characteristics are summarized in Table 1.

**Table 1:** Clinical characteristics

		Study population							CRP subpopulation						
		M	SD	Mdn	Min	Max	*n*	%	M	SD	Mdn	Min	Max	*n*	%
Age, years		57.9	7.7	57.3	44.5	73.6	23,250	100	57.4	6.0	57.7	46.0	68.0	4,196	100
Sex	Male						8,669	37						1,652	39
	Female						14,581	63						2,544	61
Height, cm		168.5	8.8	168.0	127.0	203.0	23,250	100	168.9	8.8	168.0	144.0	201.0	4,196	100
Weight, kg		72.9	13.4	72.0	31.0	170.0	23,250	100	72.9	13.1	72.0	39.0	150.0	4,196	100
Body mass index, kg/m^2^		25.6	3.9	25.2	13.9	50.9	23,250	100	25.5	3.8	25.0	15.6	50.7	4,196	100
Waist circumference, cm		83.2	12.6	82.0	50.0	152.0	23,250	100	82.7	12.4	82.0	54.0	152.0	4,196	100
Systolic blood pressure, mm Hg		140.8	19.9	140.0	61.0	230.0	23,250	100	140.7	18.7	140.0	94.0	210.0	4,196	100
Diastolic blood pressure, mm Hg		85.3	10.0	85.0	40.0	136.0	23,250	100	86.7	9.3	86.0	58.0	130.0	4,196	100
Physical activity score		8,221	6,774	6,888	0	316,120	23,250	100	8,314	5,913	7,124	0	48,900	4,196	100
Smoking Status	Regular smoker						5,460	23						906	22
	Occasional smoker						1,050	5						194	5
	Former smoker						7,763	33						1,372	33
	Never smoker						8,977	39						1,724	41
Education level	< 9 years						9,440	41						1,879	45
	9–10 years						6,173	27						1,096	26
	11–13 years						4,188	18						696	17
	University degree						3,449	15						525	13
Living alone	No						17,598	76						3,257	78
	Yes						5,652	24						939	22
Born in Sweden	Yes						20,478	88						3,755	89
	No						2,772	12						441	11
Paleolithic Diet Fraction, %		41	11	40	0	90	23,250	100	42	11	41	7	88	4,196	100
Leukocyte count, × 10^9^/L		6.3	1.5	6.0	1.6	11.0	23,250	100	5.9	1.5	5.7	2.3	11.0	4,196	100
Neutrophil count, × 10^9^/L		3.8	1.2	3.6	0.2	8.7	23,250	100	3.6	1.1	3.5	0.6	8.6	4,196	100
Lymphocyte count, × 10^9^/L		1.9	0.6	1.9	0.5	6.0	23,250	100	1.8	0.5	1.8	0.5	5.6	4,196	100
Neutrophil/lymphocyte ratio		2.1	0.7	2.0	0.2	4.9	23,250	100	2.1	0.7	1.9	0.3	4.9	4,196	100
C-reactive protein, mg/L									1.9	1.8	1.2	0.1	10.0	4,196	100
Total cholesterol, mmol/L									6.2	1.1	6.1	3.0	11.8	4,196	100
LDL, mmol/L									4.2	1.0	4.1	1.0	9.8	4,196	100
HDL, mmol/L									1.4	0.4	1.4	0.5	3.1	4,196	100
Triglycerides, mmol/L									1.3	0.8	1.1	0.3	16.3	4,196	100
Total cholesterol/HDL ratio									4.7	1.4	4.5	1.9	13.0	4,196	100
LDL/HDL ratio									3.2	1.2	3.1	0.6	9.6	4,196	100
Triglyceride/HDL ratio									1.1	0.8	0.8	0.2	6.8	4,196	100
HbA1c, %									4.8	0.5	4.8	3.3	8.9	4,196	100
Fasting glucose, mmol/L									5.0	0.6	4.9	3.3	13.5	4,196	100
Fasting insulin, mIU									7.6	8.0	6.0	2.9	224.0	4,196	100
HOMA-IR									1.8	2.6	1.4	0.4	88.3	4,196	100

Clinical characteristics of the study population and the CRP subpopulation, both comprised of participants from the Malmö Diet and Cancer Study (MDCS) without previous coronary events, diabetes, stroke, or high-grade inflammation, and with no missing covariate data at baseline (1992–96)

BMI, Body mass index; CRP, C-reactive protein; HbA1c, Hemoglobin A1c; HDL, High-density lipoprotein cholesterol; LDL, Low-density lipoprotein cholesterol; HOMA-IR, Homeostatic model assessment of insulin resistance

Mean PDF was 41% in the study population and 42% in the subpopulation. Mean total leukocyte count was 6.3 × 10^9^/L and 5.9 × 10^9^/L in the study population and subpopulation, respectively. Mean NLR was 2.1 in both populations and mean CRP was 1.9 mg/L in the subpopulation. Daily food intake at baseline is summarized in Table 2.

**Table 2:** Daily Food Intake

	Study population (*n* = 23,250)				CRP subpopulation (*n* = 4,196)			
	Mdn	Min	Max	IQR	Mdn	Min	Max	IQR
Paleolithic diet fraction, %	40	0	90	15	41	7	88	15
Total food group weight, g/day	1,689	330	5,242	620	1,735	488	4,371	629
Total food group energy, kcal	2,180	516	8,396	798	2,224	627	5,879	825
Total food group energy per gram, kcal	1	0.5	3	0.3	1	0.6	2	0.3
Paleolithic food groups, g	668	0	4,089	290	700	138	2,060	294
Vegetables, g	146	0	1,177	110	162	1	933	119
Fruits, g	168	0	2,782	152	182	0	1,312	153
Potatoes, g	109	0	1,447	84	110	0	862	91
Eggs, g	19	0	245	22	19	0	173	22
Meat^a^, g	94	0	593	60	96	0	470	62
Fish, g	39	0	527	41	40	0	505	41
Rapeseed and olive oil, g	0	0	74	0	0	0	26	0
Nuts, g	0	0	147	2	0	0	75	2
Wine, g	21	0	1,127	71	21	0	764	71
Non-Paleolithic food groups, g	999	75	4,182	512	1,011	129	3,417	513
Legumes, g	0	0	520	14	0	0	206	18
Juice, g	1	0	1,371	100	1	0	1,371	100
Meat products^b^, g	24	0	341	35	25	0	293	37
Milk and milk products, g	395	0	3,339	315	406	0	2,200	315
Sweet beverages, g	9	0	3,000	94	6	0	1,714	86
Cereal grains (including rice), g	132	0	1,114	81	139	1	1,114	88
Fats, Oils, and Margarines, g	30	0	209	31	32	0	205	33
Bakery sweets, g	65	0	715	57	66	0	715	58
Jam, g	12	0	205	20	13	0	205	21
Sauces and soups, g	3	0	1,000	10	3	0	361	10
Beer, g	83	0	3,143	193	86	0	1,961	190
Spirits, g	0	0	550	7	0	0	236	7
Remainder miscellaneous, g	4	0	692	4	4	0	131	4

Daily food group intake in the study population and the CRP subpopulation, both comprised of participants from the Malmö Diet and Cancer Study (MDCS) without previous coronary events, diabetes, stroke, or high-grade inflammation, and with no missing covariate data at baseline (1992–96). Non-energy containing beverages excluded

CRP, C-reactive protein. Total food group energy’ refers to the overall daily energy intake from all foods combined. Disaggregated energy values for individual food groups are not available in the MDCS dataset

^a^Pork, beef, lamb, game meat, poultry, and pure offal. ^b^ Offal as a mixed product or spread, and sausage

**Fig. 2:**
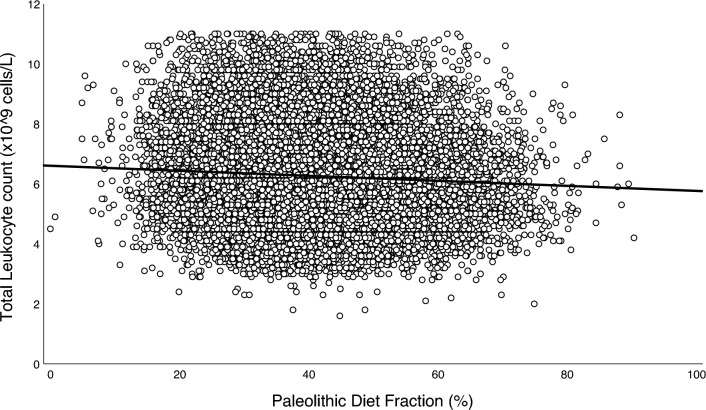
Association between Paleolithic Diet Fraction and total leukocyte count. *Note.* Association between Paleolithic Diet Fraction and total leukocyte count in the study population (*n* = 23,250) comprised of participants from the Malmö Diet and Cancer Study (MDCS) without previous coronary events, diabetes, stroke, or high-grade inflammation, and with no missing covariate data at baseline (1992–96). The solid line depicts the fully adjusted linear regression fit (*y* = 6.603 − 0.008 × *x*, *p* < 0.001)

**Fig. 3:**
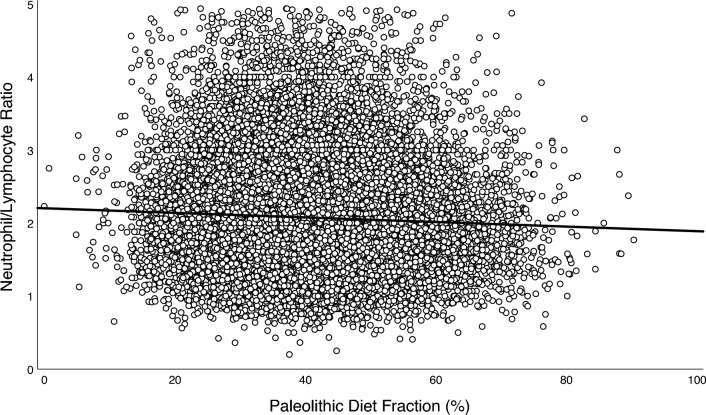
Association between Paleolithic Diet Fraction and neutrophil-to-lymphocyte ratio. *Note.* Association between Paleolithic Diet Fraction and neutrophil-to-lymphocyte ratio in the study population (*n* = 23,250) comprised of participants from the Malmö Diet and Cancer Study (MDCS) without previous coronary events, diabetes, stroke, or high-grade inflammation, and with no missing covariate data at baseline (1992–96). The solid line depicts the fully adjusted linear regression fit (*y* = 2.203 − 0.003 × *x*, *p* < 0.001)

**Fig. 4:**
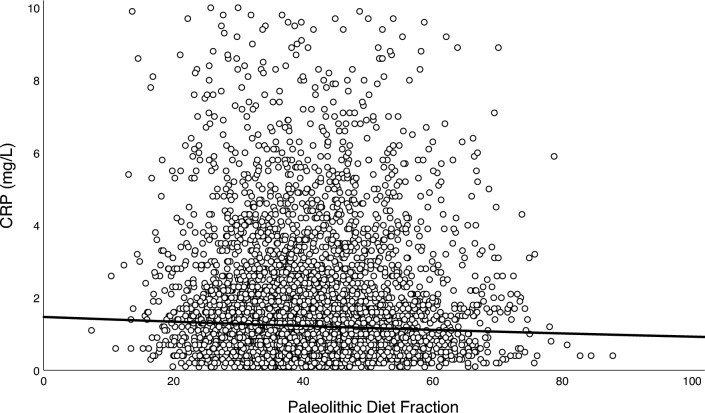
Association between Paleolithic Diet Fraction and c-reactive protein. *Note.* Association between Paleolithic Diet Fraction and C-reactive protein (CRP) in the CRP subpopulation (*n* = 4196) comprised of participants from the Malmö Diet and Cancer Study (MDCS) without previous coronary events, diabetes, stroke, or high-grade inflammation, and with no missing covariate data at baseline (1992–96). The solid line depicts the fully adjusted regression fit, back-transformed from the natural logarithm of CRP [*y* = exp(0.385 − 0.005 × *x*), *p* < 0.001]

Highly significant (*p* < 0.001) but weak positive correlations were found among all biomarkers of inflammation, using Spearman’s correlation coefficient (*r*_*s*_): total leukocyte count and NLR (*r*_*s*_ = 0.263), total leukocyte count and CRP (*r*_*s*_ = 0.262), and NLR and CRP (*r*_*s*_ = 0.062). PDF was highly significantly and inversely associated with all biomarkers of inflammation in both simple and adjusted multiple linear regressions (*p* < 0.001; Table 3 and Figs. 2, 3 and 4). Sensitivity analyses stratified by sex, age, and PDF found similar inverse associations across all strata, albeit with a significant interaction between PDF and age for NLR (*p* = 0.004, Table S1). Exploratory analyses of food groups in fully adjusted models showed that vegetables, fruits, and fish were negatively associated with SLGCI biomarkers, whereas meat products, milk and milk products, bakery sweets, and sweet beverages were positively associated (Table S2).

**Table 3:** Association between Paleolithic Diet Fraction and inflammatory biomarkers

Biomarker	Model	*n*	*B* for constant	*B*	*SE* Coeff	ß	*t*	*p*	Adj *R*^*2*^ for model
Total leukocyte count, × 10^9^/L		23,250							
	Non-adjusted		6.749	− 0.012	0.001	− 0.090	− 13.757	< 0.001	0.008
	Age and sex adjusted			− 0.013	0.001	− 0.100	− 14.895	< 0.001	0.010
	Fully adjusted*			− 0.008	0.001	− 0.063	− 10.037	< 0.001	0.158
Neutrophil-to-lymphocyte ratio		23,250							
	Non-adjusted		2.272	− 0.005	0.000	− 0.075	− 11.409	< 0.001	0.006
	Age and sex adjusted			− 0.004	0.000	− 0.060	− 8.924	< 0.001	0.010
	Fully adjusted*			− 0.003	0.000	− 0.048	− 7.139	< 0.001	0.019
Ln C-reactive protein		4,196							
	Non-adjusted		0.395	− 0.005	0.001	− 0.057	− 3.675	< 0.001	0.003
	Age and sex adjusted			− 0.005	0.001	− 0.060	− 3.790	< 0.001	0.012
	Fully adjusted*			− 0.005	0.001	− 0.054	− 3.555	< 0.001	0.114

Association between Paleolithic Diet Fraction (%) and inflammatory biomarkers assessed using simple and multivariable linear regression models. The study population and the CRP subpopulation were both comprised of participants from the Malmö Diet and Cancer Study (MDCS) without previous coronary events, diabetes, stroke, or high-grade inflammation, and with no missing covariate data at baseline (1992–96)

*Adjusted for age, sex, physical activity level, body mass index, smoking status, education level, living alone, born in Sweden, season of dietary data collection, and dietary method version

## Discussion

This study investigated the associations between PDF, a measure estimating the proportion of dietary intake derived from food groups consistent with the Paleolithic diet, and biomarkers of SLGCI in a large Swedish cohort (the Malmö Diet and Cancer Study, MDCS). The main finding was that PDF was consistently and inversely associated with all biomarkers of SLGCI, even after adjusting for potential confounders. In sensitivity analyses, the inverse association between PDF and biomarkers of SLGCI was broadly consistent across sex, age, and PDF strata. Notably, for NLR, there was a significant interaction between PDF and age, with the greatest explanatory power of the model among older participants, consistent with the concept of inflammaging, whereby SLGCI becomes more prevalent with age [1]. However, no such age interaction was observed for total leukocyte count or CRP. Instead, for CRP, the inverse association with PDF was greatest and only significant in the PDF tertile with the lowest PDF values, suggesting a potential diminishing returns effect whereby those with the lowest baseline PDF values stand to gain the most from dietary shifts toward a Paleolithic pattern. These findings align with previous research on the effects of the Paleolithic diet on CRP in interventional and observational studies [31, 37, 48]. Nevertheless, a recent umbrella review found no consistent inverse association between a Paleolithic diet and CRP [49]. Only a limited number of Paleolithic dietary trials have examined inflammatory biomarkers [31, 37], and, among these, not all had inflammation as the primary outcome [33, 50–54], which can reduce their ability to detect significant changes in inflammatory biomarkers. Furthermore, Paleolithic diets, although similar in terms of excluded foods, may vary in their composition of included foods known to decrease inflammation, such as fish, fruits, and vegetables [23, 26]. In exploratory analyses, food group associations with SLGCI biomarkers showed a coherent pattern: Paleolithic food groups, particularly vegetables, fruits, and fish, were negatively associated, whereas non-Paleolithic food groups, including meat products and milk and milk products, were positively associated. This pattern supports the rationale and design of PDF. Moreover, all standardized coefficients for individual food groups were smaller than those for PDF, highlighting the usefulness of employing a dietary pattern measure such as PDF when assessing diet-SLGCI associations. Regarding total leukocyte count and NLR, no previous study assessing the effect of the Paleolithic diet on those biomarkers was found, thus precluding direct comparison with our results. As for other dietary patterns, the findings of this study concur with previous studies suggesting that emphasizing nutrient-dense foods while minimizing salt, refined sugars, trans fatty acids, and ultra-processed foods can lower biomarkers of systemic inflammation, including CRP [23–25, 55]. The inverse associations between PDF and inflammatory biomarkers, coupled with the previously documented association between cardiometabolic health and both PDF and biomarkers of inflammation [2, 8, 34], suggest that SLGCI could be a mediator between PDF and the incidence of cardiometabolic disease and mortality in the MDCS. The hypothesis that SLGCI could be a mediator between lifestyle factors, such as diet and cardiometabolic disease risk, is supported by comparisons of median levels of inflammatory biomarkers between populations, where the median levels of CRP (1.2 mg/L), total leukocyte count (6.0 × 10^9^/L), and NLR (2.0) of the MDCS population are comparable to those observed in other industrialized country cohorts [14, 56–59]. By contrast, CRP levels were lower in traditional populations with low cardiometabolic risk, such as the Melanesian horticulturalists from Kitava in Papua New Guinea [60], the Shuar forager-horticulturalists from the Ecuadorian Amazon [61], and the subsistence-agriculturalists from rural Ghana [62].

In the present study, mean PDF was roughly 40%, which is similar to what has been observed for non-Paleolithic diets in previous interventional and observational studies [38, 39, 48]. In contrast, mean PDF for the Paleolithic diet in previous RCTs was around 80% [38, 39]. Consuming a Paleolithic diet with a PDF of 80% instead of a non-Paleolithic diet with a PDF of 40% would, based on the findings of this study, be associated with mean differences of approximately − 0.33 × 10^9^/L in total leukocyte count, −0.13 in NLR, and −0.21 mg/L in CRP. Based on previously assessed associations between SLGCI and cardiometabolic disease incidence in MDCS [2], these mean differences in inflammatory biomarkers would be associated with between 0.6–3.6% lower adjusted cardiometabolic disease risks. Applying such lower disease risks to the whole previously assessed MDCS population of 25,969 individuals would translate to around 200 fewer cardiometabolic disease incidences out of 8,367 cardiometabolic disease incidences after 17.7 years [2].

### Strengths and limitations

A major strength of this study is its large sample size and the use of a validated dietary assessment method, which allowed a precise estimation of PDF. Additionally, the inclusion of multiple inflammatory biomarkers provides a more comprehensive assessment of SLGCI. Moreover, the fact that CRP was measured approximately four months after total leukocyte count and NLR, yet still showed significant correlations with them, reinforces their interpretation as biomarkers of chronic rather than transient inflammation. The high quality and comprehensiveness of baseline data collection in the MDCS, together with adjustment for various potential confounders, strengthens the robustness of the findings.

However, several limitations should be noted. First, dietary intake was assessed at a single time point, precluding the ability to examine longitudinal changes in diet. Second, although the MDCS is a prospective cohort study, the present analysis is cross‑sectional, examining associations between baseline dietary data and biomarkers measured at or near baseline; as such, it limits the ability to establish temporal relationships and weakens causal inference. This is particularly relevant for CRP, which was measured approximately four months after baseline examinations, potentially weakening its association with variables assessed at baseline, including PDF. This time gap may have introduced the possibility that changes in inflammatory status due to factors unrelated to diet (e.g., psychological stress [63], changes in sleep patterns [64, 65], or physical activity fluctuations [66]) could have influenced CRP levels. Nonetheless, CRP remained significantly, albeit weakly, correlated with both total leukocyte count and NLR despite being measured later, indicating stability of SLGCI over time and supporting the reliability of its associations with PDF. Third, the absolute magnitude of the observed associations was modest, which may raise questions about their clinical relevance. Nevertheless, as previously illustrated, even small differences in SLGCI biomarkers may translate into meaningful reductions in disease burden at the population level. However, achieving the increase in PDF needed to produce even small differences in SLGCI biomarkers (e.g., from 40% to 80%) may be difficult for many individuals in real-world settings, which should be considered when assessing the feasibility and potential impact of such dietary changes at scale. Fourth, residual confounding cannot be ruled out despite adjustments for potential confounders. This includes the possibility of unmeasured factors such as health-seeking behaviors, whereby individuals with higher PDF may also engage in other health-promoting practices (e.g., higher preventive healthcare use, better adherence to medical advice) that could independently lower systemic inflammation. Moreover, participation in the MDCS itself likely reflects a certain degree of health interest and motivation, since those who agreed to participate may differ systematically from non-participants in ways that could influence both diet and inflammation. Fifth, the study population consisted predominantly of middle-aged Swedish individuals, the majority being women (63%) in an urban setting, which may limit the generalizability of the findings to other populations with different ethnic backgrounds, cultural contexts, and dietary habits. Finally, although the dietary assessment method employed in the MDCS was specifically developed for this cohort and has demonstrated good validity and reproducibility in previous studies [34, 44, 45], it nonetheless relies on self-reported data, which is inherently susceptible to measurement error and recall bias [67–69]. The use of a multi-method approach, which included a 7-day food record, a 168-item food frequency questionnaire, and a structured interview with a trained dietician, likely mitigated these sources of error to some extent. However, residual bias cannot be excluded, particularly for food items with high day-to-day variability [70] or socially desirable reporting patterns [71].

### Implications for future research

Future research should focus on longitudinal observational studies and interventional trials, specifically examining the effects of the Paleolithic diet and PDF on inflammatory biomarkers. In particular, well-powered RCTs should prioritize inflammation as a primary outcome and assess biomarkers including CRP, total leukocyte count, NLR, and additional SLGCI biomarkers such as cytokines. Additionally, the biological mechanisms underlying the association between PDF and systemic inflammation should be explored. Further research in more ethnically diverse populations with varied dietary habits and cultural contexts is warranted to enhance the generalizability of the findings and better assess the potential of PDF as a dietary marker for reducing SLGCI and associated disease risks.

## Conclusion

PDF and SLGCI were inversely associated, suggesting that SLGCI could mediate the effect of PDF on cardiometabolic disease and mortality. However, given the cross-sectional design, the small absolute magnitude of associations, and the potential influence of residual confounding, these findings should be interpreted with caution. Further longitudinal and interventional studies in diverse populations are warranted to clarify causality, identify contributing dietary components, and assess the clinical relevance of modifying PDF to reduce SLGCI. These findings, if confirmed, could inform dietary recommendations aimed at mitigating low-grade inflammation and improving cardiometabolic health.

## Supplementary Information

Below is the link to the electronic supplementary material.

Supplementary file1 (PDF 76 KB)

Supplementary file2 (PDF 80 KB)

## Data Availability

Data described in the manuscript can be made available upon request pending application and approval by the chair of the steering committee for the cohort.

## Abbreviations

BMI: Body mass index
CRP: C-reactive protein
HbA1c: Hemoglobin A1c
PDF: Paleolithic diet fraction
MDCS: Malmö diet and cancer study
ICD: International classification of diseases
NLR: Neutrophil-to-lymphocyte ratio
RCTs: Randomized controlled trials
SLGCI: Systemic low-grade chronic inflammation
SBP and DBP: Systolic and diastolic blood pressure

## References

[1] Furman D, Campisi J, Verdin E et al (2019) Chronic inflammation in the etiology of disease across the life span. Nat Med 25:1822–1832. 10.1038/s41591-019-0675-0 31806905 10.1038/s41591-019-0675-0 PMC7147972

[2] Bao X, Borné Y, Johnson L et al (2018) Comparing the inflammatory profiles for incidence of diabetes mellitus and cardiovascular diseases: a prospective study exploring the ‘common soil’ hypothesis. Cardiovasc Diabetol 17:87. 10.1186/s12933-018-0733-9 29895294 10.1186/s12933-018-0733-9 PMC5996509

[3] Speer T, Dimmeler S, Schunk SJ et al (2022) Targeting innate immunity-driven inflammation in CKD and cardiovascular disease. Nat Rev Nephrol 18:762–778. 10.1038/s41581-022-00621-9 36064794 10.1038/s41581-022-00621-9

[4] Mensah GA, Fuster V, Murray CJL et al (2023) Global burden of cardiovascular diseases and risks, 1990–2022. J Am Coll Cardiol 82:2350–2473. 10.1016/j.jacc.2023.11.007 38092509 10.1016/j.jacc.2023.11.007 PMC7615984

[5] Ong KL, Stafford LK, McLaughlin SA et al (2023) Global, regional, and national burden of diabetes from 1990 to 2021, with projections of prevalence to 2050: a systematic analysis for the Global Burden of Disease Study 2021. Lancet 402:203–234. 10.1016/S0140-6736(23)01301-6 37356446 10.1016/S0140-6736(23)01301-6 PMC10364581

[6] Calder PC, Ahluwalia N, Albers R et al (2013) A consideration of biomarkers to be used for evaluation of inflammation in human nutritional studies. Br J Nutr 109(Suppl 1):S1-34. 10.1017/S0007114512005119 23343744 10.1017/S0007114512005119

[7] Ridker PM (2016) A test in context: high-sensitivity C-reactive protein. J Am Coll Cardiol 67:712–723. 10.1016/j.jacc.2015.11.037 26868696 10.1016/j.jacc.2015.11.037

[8] Zia E, Melander O, Björkbacka H et al (2012) Total and differential leucocyte counts in relation to incidence of stroke subtypes and mortality: a prospective cohort study. J Intern Med 272:298–304. 10.1111/j.1365-2796.2012.02526.x 22303818 10.1111/j.1365-2796.2012.02526.x

[9] Babio N, Ibarrola-Jurado N, Bulló M et al (2013) White blood cell counts as risk markers of developing metabolic syndrome and its components in the Predimed study. PLoS ONE 8:e58354. 10.1371/journal.pone.0058354 23526980 10.1371/journal.pone.0058354 PMC3602299

[10] Avery EF, Kleynhans JN, Ledergerber B et al (2023) Leukocyte count and coronary artery disease events in people with human immunodeficiency virus: a longitudinal study. Clin Infect Dis 76:1969–1979. 10.1093/cid/ciad033 36688465 10.1093/cid/ciad033 PMC10249993

[11] Wang Q, Guo Q, Zhou L et al (2022) Associations of baseline and changes in leukocyte counts with incident cardiovascular events: the Dongfeng-Tongji cohort study. J Atheroscler Thromb 29:1040–1058. 10.5551/jat.62970 34305075 10.5551/jat.62970 PMC9252621

[12] Buonacera A, Stancanelli B, Colaci M, Malatino L (2022) Neutrophil to lymphocyte ratio: an emerging marker of the relationships between the immune system and diseases. Int J Mol Sci 23:3636. 10.3390/ijms23073636 35408994 10.3390/ijms23073636 PMC8998851

[13] Liu C-C, Ko H-J, Liu W-S et al (2019) Neutrophil-to-lymphocyte ratio as a predictive marker of metabolic syndrome. Medicine 98:e17537. 10.1097/MD.0000000000017537 31651856 10.1097/MD.0000000000017537 PMC6824790

[14] Chen HL, Wu C, Cao L et al (2024) The association between the neutrophil-to-lymphocyte ratio and type 2 diabetes mellitus: a cross-sectional study. BMC Endocr Disord 24:107. 10.1186/s12902-024-01637-x 38982402 10.1186/s12902-024-01637-x PMC11232124

[15] Wang Q-C, Wang Z-Y (2023) Comparative analysis of neutrophil-to-lymphocyte ratio and remnant cholesterol in predicting cardiovascular events and mortality in general adult population. Sci Rep 13:22362. 10.1038/s41598-023-49403-8 38102174 10.1038/s41598-023-49403-8 PMC10724289

[16] Song M, Graubard BI, Rabkin CS, Engels EA (2021) Neutrophil-to-lymphocyte ratio and mortality in the United States general population. Sci Rep 11:464. 10.1038/s41598-020-79431-7 33431958 10.1038/s41598-020-79431-7 PMC7801737

[17] Li X, Liu M, Wang G (2024) The neutrophil–lymphocyte ratio is associated with all-cause and cardiovascular mortality in cardiovascular patients. Sci Rep 14:26692. 10.1038/s41598-024-76836-6 39496711 10.1038/s41598-024-76836-6 PMC11535400

[18] Bhattacharya S, Munshi C (2023) Biological significance of C-reactive protein, the ancient acute phase functionary. Front Immunol 14:1238411. 10.3389/fimmu.2023.1238411 37860004 10.3389/fimmu.2023.1238411 PMC10582692

[19] Wang X, Bao W, Liu J et al (2013) Inflammatory markers and risk of type 2 diabetes: a systematic review and meta-analysis. Diabetes Care 36:166–175. 10.2337/dc12-0702 23264288 10.2337/dc12-0702 PMC3526249

[20] Li Y, Zhong X, Cheng G et al (2017) Hs-CRP and all-cause, cardiovascular, and cancer mortality risk: a meta-analysis. Atherosclerosis 259:75–82. 10.1016/j.atherosclerosis.2017.02.003 28327451 10.1016/j.atherosclerosis.2017.02.003

[21] Ni P, Yu M, Zhang R et al (2020) Dose-response association between C-reactive protein and risk of all-cause and cause-specific mortality: a systematic review and meta-analysis of cohort studies. Ann Epidemiol 51:20-27.e11. 10.1016/j.annepidem.2020.07.005 32702432 10.1016/j.annepidem.2020.07.005

[22] Minihane AM, Vinoy S, Russell WR et al (2015) Low-grade inflammation, diet composition and health: current research evidence and its translation. Br J Nutr 114:999–1012. 10.1017/S0007114515002093 26228057 10.1017/S0007114515002093 PMC4579563

[23] Yu X, Pu H, Voss M (2024) Overview of anti-inflammatory diets and their promising effects on non-communicable diseases. Br J Nutr 132:898–918. 10.1017/S0007114524001405 39411832 10.1017/S0007114524001405 PMC11576095

[24] Neale EP, Batterham MJ, Tapsell LC (2016) Consumption of a healthy dietary pattern results in significant reductions in C-reactive protein levels in adults: a meta-analysis. Nutr Res 36:391–401. 10.1016/j.nutres.2016.02.009 27101757 10.1016/j.nutres.2016.02.009

[25] Pickworth CK, Deichert DA, Corroon J, Bradley RD (2019) Randomized controlled trials investigating the relationship between dietary pattern and high-sensitivity C-reactive protein: a systematic review. Nutr Rev 77:363–375. 10.1093/nutrit/nuz003 31222367 10.1093/nutrit/nuz003

[26] Hosseini B, Berthon BS, Saedisomeolia A et al (2018) Effects of fruit and vegetable consumption on inflammatory biomarkers and immune cell populations: a systematic literature review and meta-analysis. Am J Clin Nutr 108:136–155. 10.1093/ajcn/nqy082 29931038 10.1093/ajcn/nqy082

[27] Jiang R, Jacobs DR, Mayer-Davis E et al (2006) Nut and seed consumption and inflammatory markers in the multi-ethnic study of atherosclerosis. Am J Epidemiol 163:222–231. 10.1093/aje/kwj033 16357111 10.1093/aje/kwj033

[28] Yu Z, Malik VS, Keum N et al (2016) Associations between nut consumption and inflammatory biomarkers. Am J Clin Nutr 104:722–728. 10.3945/ajcn.116.134205 27465378 10.3945/ajcn.116.134205 PMC4997300

[29] Zampelas A, Panagiotakos DB, Pitsavos C et al (2005) Fish consumption among healthy adults is associated with decreased levels of inflammatory markers related to cardiovascular disease: the ATTICA study. J Am Coll Cardiol 46:120–124. 10.1016/j.jacc.2005.03.048 15992645 10.1016/j.jacc.2005.03.048

[30] Hart MJ, Torres SJ, McNaughton SA, Milte CM (2021) Dietary patterns and associations with biomarkers of inflammation in adults: a systematic review of observational studies. Nutr J 20:24. 10.1186/s12937-021-00674-9 33712009 10.1186/s12937-021-00674-9 PMC7955619

[31] Sohouli MH, Fatahi S, Lari A et al (2022) The effect of paleolithic diet on glucose metabolism and lipid profile among patients with metabolic disorders: a systematic review and meta-analysis of randomized controlled trials. Crit Rev Food Sci Nutr 62:4551–4562. 10.1080/10408398.2021.1876625 33492173 10.1080/10408398.2021.1876625

[32] Lindeberg S, Jönsson T, Granfeldt Y et al (2007) A palaeolithic diet improves glucose tolerance more than a Mediterranean-like diet in individuals with ischaemic heart disease. Diabetologia 50:1795–1807. 10.1007/s00125-007-0716-y 17583796 10.1007/s00125-007-0716-y

[33] Jönsson T, Granfeldt Y, Ahrén B et al (2009) Beneficial effects of a Paleolithic diet on cardiovascular risk factors in type 2 diabetes: a randomized cross-over pilot study. Cardiovasc Diabetol 8:35. 10.1186/1475-2840-8-35 19604407 10.1186/1475-2840-8-35 PMC2724493

[34] Rydhög B, Carrera-Bastos P, Granfeldt Y et al (2024) Inverse association between Paleolithic diet fraction and mortality and incidence of cardiometabolic disease in the prospective Malmö diet and cancer study. Eur J Nutr 63:501–512. 10.1007/s00394-023-03279-6 38078965 10.1007/s00394-023-03279-6 PMC10899283

[35] Whalen KA, Judd S, McCullough ML et al (2017) Paleolithic and Mediterranean diet pattern scores are inversely associated with all-cause and cause-specific mortality in adults. J Nutr 147:612–620. 10.3945/jn.116.241919 28179490 10.3945/jn.116.241919 PMC5368578

[36] Manheimer EW, van Zuuren EJ, Fedorowicz Z, Pijl H (2015) Paleolithic nutrition for metabolic syndrome: systematic review and meta-analysis. Am J Clin Nutr 102:922–932. 10.3945/ajcn.115.113613 26269362 10.3945/ajcn.115.113613 PMC4588744

[37] Ghaedi E, Mohammadi M, Mohammadi H et al (2019) Effects of a paleolithic diet on cardiovascular disease risk factors: a systematic review and meta-analysis of randomized controlled trials. Adv Nutr 10:634–646. 10.1093/advances/nmz007 31041449 10.1093/advances/nmz007 PMC6628854

[38] Rydhög B, Granfeldt Y, Frassetto L et al (2019) Assessing compliance with Paleolithic diet by calculating Paleolithic diet fraction as the fraction of intake from Paleolithic food groups. Clin Nutr Exp 25:29–35. 10.1016/j.yclnex.2019.03.002

[39] Rydhög B, Granfeldt Y, Sundquist K, Jönsson T (2021) Paleolithic diet fraction in post hoc data analysis of a randomized cross-over study comparing Paleolithic diet with diabetes diet. Clin Nutr Open Sci 38:73–80. 10.1016/j.nutos.2021.07.001

[40] Manjer J, Carlsson S, Elmståhl S et al (2001) The Malmö diet and cancer study: representativity, cancer incidence and mortality in participants and non-participants. Eur J Cancer Prev 10:489–499. 10.1097/00008469-200112000-00003 11916347 10.1097/00008469-200112000-00003

[41] Riley LK, Rupert J (2015) Evaluation of patients with leukocytosis. Am Fam Physician 92:1004–1011 26760415

[42] Gürol G, Çiftci İH, Terizi HA et al (2015) Are there standardized cutoff values for neutrophil-lymphocyte ratios in bacteremia or sepsis? J Microbiol Biotechnol 25:521–525. 10.4014/jmb.1408.08060 25341467 10.4014/jmb.1408.08060

[43] Hedblad B, Nilsson P, Janzon L, Berglund G (2000) Relation between insulin resistance and carotid intima-media thickness and stenosis in non-diabetic subjects. Results from a cross-sectional study in Malmö, Sweden. Diabet Med 17:299–307. 10.1046/j.1464-5491.2000.00280.x 10821297 10.1046/j.1464-5491.2000.00280.x

[44] Callmer E, Riboli E, Saracci R et al (1993) Dietary assessment methods evaluated in the Malmö food study. J Intern Med 233:53–57. 10.1111/j.1365-2796.1993.tb00648.x 8429287 10.1111/j.1365-2796.1993.tb00648.x

[45] Wirfält E, Mattisson I, Johansson U et al (2002) A methodological report from the Malmö diet and cancer study: development and evaluation of altered routines in dietary data processing. Nutr J 1:3. 10.1186/1475-2891-1-3 12537595 10.1186/1475-2891-1-3 PMC149436

[46] WHO Consultation on Obesity (1999: Geneva S, Organization WH (2000) Obesity : preventing and managing the global epidemic : report of a WHO consultation. p 252 11234459

[47] Bergwall S, Acosta S, Ramne S et al (2021) Leisure-time physical activities and the risk of cardiovascular mortality in the Malmö diet and cancer study. BMC Public Health 21:1948. 10.1186/s12889-021-11972-6 34702239 10.1186/s12889-021-11972-6 PMC8549319

[48] Whalen KA, McCullough ML, Flanders WD et al (2016) Paleolithic and Mediterranean diet pattern scores are inversely associated with biomarkers of inflammation and oxidative balance in adults. J Nutr 146:1217–1226. 10.3945/jn.115.224048 27099230 10.3945/jn.115.224048 PMC4877627

[49] Tran DQ, Di Nguyen K, Quynh Chi VT, Nguyen HTH (2024) Evaluating the effects of dietary patterns on circulating C-reactive protein levels in the general adult population: an umbrella review of meta-analyses of interventional and observational studies. Br J Nutr 132:783–793. 10.1017/S0007114524001648 39364652 10.1017/S0007114524001648

[50] Osterdahl M, Kocturk T, Koochek A, Wändell PE (2008) Effects of a short-term intervention with a paleolithic diet in healthy volunteers. Eur J Clin Nutr 62:682–685. 10.1038/sj.ejcn.1602790 17522610 10.1038/sj.ejcn.1602790

[51] Mellberg C, Sandberg S, Ryberg M et al (2014) Long-term effects of a Palaeolithic-type diet in obese postmenopausal women: a 2-year randomized trial. Eur J Clin Nutr 68:350–357. 10.1038/ejcn.2013.290 24473459 10.1038/ejcn.2013.290 PMC4216932

[52] Boers I, Muskiet FA, Berkelaar E et al (2014) Favourable effects of consuming a Palaeolithic-type diet on characteristics of the metabolic syndrome: a randomized controlled pilot-study. Lipids Health Dis 13:160. 10.1186/1476-511X-13-160 25304296 10.1186/1476-511X-13-160 PMC4210559

[53] Genoni A, Lyons-Wall P, Lo J, Devine A (2016) Cardiovascular, metabolic effects and dietary composition of ad-libitum Paleolithic vs. Australian guide to healthy eating diets: a 4-week randomised trial. Nutrients 8:314. 10.3390/nu8050314 27223304 10.3390/nu8050314 PMC4882726

[54] Irish AK, Erickson CM, Wahls TL et al (2017) Randomized control trial evaluation of a modified Paleolithic dietary intervention in the treatment of relapsing-remitting multiple sclerosis: a pilot study. Degener Neurol Neuromuscul Dis 7:1–18. 10.2147/DNND.S116949 30050374 10.2147/DNND.S116949 PMC6053098

[55] Norde MM, Collese TS, Giovannucci E, Rogero MM (2021) A posteriori dietary patterns and their association with systemic low-grade inflammation in adults: a systematic review and meta-analysis. Nutr Rev 79:331–350. 10.1093/nutrit/nuaa010 32417914 10.1093/nutrit/nuaa010

[56] Emerging Risk Factors Collaboration, Kaptoge S, Di Angelantonio E et al (2010) C-reactive protein concentration and risk of coronary heart disease, stroke, and mortality: an individual participant meta-analysis. Lancet 375:132–140. 10.1016/S0140-6736(09)61717-7 20031199 10.1016/S0140-6736(09)61717-7 PMC3162187

[57] Rolver MG, Emanuelsson F, Nordestgaard BG, Benn M (2024) Contributions of elevated CRP, hyperglycaemia, and type 2 diabetes to cardiovascular risk in the general population: observational and Mendelian randomization studies. Cardiovasc Diabetol 23:165. 10.1186/s12933-024-02207-0 38730445 10.1186/s12933-024-02207-0 PMC11088022

[58] Kato K, Otsuka T, Saiki Y et al (2019) Association between elevated C-reactive protein levels and prediabetes in adults, particularly impaired glucose tolerance. Can J Diabetes 43:40-45.e2. 10.1016/j.jcjd.2018.03.007 30026044 10.1016/j.jcjd.2018.03.007

[59] Keller MF, Reiner AP, Okada Y et al (2014) Trans-ethnic meta-analysis of white blood cell phenotypes. Hum Mol Genet 23:6944–6960. 10.1093/hmg/ddu401 25096241 10.1093/hmg/ddu401 PMC4245044

[60] Carrera-Bastos P, Fontes-Villalba M, Gurven M et al (2020) C-reactive protein in traditional Melanesians on Kitava. BMC Cardiovasc Disord 20:524. 10.1186/s12872-020-01812-7 33334321 10.1186/s12872-020-01812-7 PMC7745357

[61] McDade TW, Tallman PS, Madimenos FC et al (2012) Analysis of variability of high sensitivity C-reactive protein in lowland Ecuador reveals no evidence of chronic low-grade inflammation. Am J Hum Biol 24:675–681. 10.1002/ajhb.22296 22639072 10.1002/ajhb.22296

[62] Eriksson UK, van Bodegom D, May L et al (2013) Low C-reactive protein levels in a traditional West-African population living in a malaria endemic area. PLoS ONE 8:e70076. 10.1371/journal.pone.0070076 23922912 10.1371/journal.pone.0070076 PMC3724900

[63] Marsland AL, Walsh C, Lockwood K, John-Henderson NA (2017) The effects of acute psychological stress on circulating and stimulated inflammatory markers: a systematic review and meta-analysis. Brain Behav Immun 64:208–219. 10.1016/j.bbi.2017.01.011 28089638 10.1016/j.bbi.2017.01.011 PMC5553449

[64] Vgontzas AN, Zoumakis E, Bixler EO et al (2004) Adverse effects of modest sleep restriction on sleepiness, performance, and inflammatory cytokines. J Clin Endocrinol Metab 89:2119–2126. 10.1210/jc.2003-031562 15126529 10.1210/jc.2003-031562

[65] Meier-Ewert HK, Ridker PM, Rifai N et al (2004) Effect of sleep loss on C-reactive protein, an inflammatory marker of cardiovascular risk. J Am Coll Cardiol 43:678–683. 10.1016/j.jacc.2003.07.050 14975482 10.1016/j.jacc.2003.07.050

[66] Fedewa MV, Hathaway ED, Ward-Ritacco CL (2017) Effect of exercise training on C reactive protein: a systematic review and meta-analysis of randomised and non-randomised controlled trials. Br J Sports Med 51:670–676. 10.1136/bjsports-2016-095999 27445361 10.1136/bjsports-2016-095999

[67] Barnard J, Tapsell L, Davies P et al (2002) Relationship of high energy expenditure and variation in dietary intake with reporting accuracy on 7 day food records and diet histories in a group of healthy adult volunteers. Eur J Clin Nutr 56:358–367. 10.1038/sj.ejcn.1601341 11965513 10.1038/sj.ejcn.1601341

[68] Althubaiti A (2016) Information bias in health research: definition, pitfalls, and adjustment methods. J Multidiscip Healthc 9:211–217. 10.2147/JMDH.S104807 27217764 10.2147/JMDH.S104807 PMC4862344

[69] Nunes CL, Jesus F, Oliveira MV et al (2024) The impact of body composition on the degree of misreporting of food diaries. Eur J Clin Nutr 78:209–216. 10.1038/s41430-023-01382-9 38087045 10.1038/s41430-023-01382-9

[70] Bailey RL (2021) Overview of dietary assessment methods for measuring intakes of foods, beverages, and dietary supplements in research studies. Curr Opin Biotechnol 70:91–96. 10.1016/j.copbio.2021.02.007 33714006 10.1016/j.copbio.2021.02.007 PMC8338737

[71] Taylor RM, Haslam RL, Burrows TL et al (2019) Issues in measuring and interpreting diet and its contribution to obesity. Curr Obes Rep 8:53–65. 10.1007/s13679-019-00336-2 30877574 10.1007/s13679-019-00336-2

