# Supplementary material for: Association between Paleolithic diet fraction and systemic low-grade chronic inflammation in the Malmö diet and cancer study cohort
Source: Eur J Nutr. 2025 Nov 12;64(8):317. doi: 10.1007/s00394-025-03838-z (PMC12612027; doi:10.1007/s00394-025-03838-z)
Supplement: Supplementary file 1 — Supplementary file1 (PDF 76 KB) [file 394_2025_3838_MOESM1_ESM.pdf]

Table S1

Association Between Paleolithic Diet Fraction (PDF) and Inflammatory Biomarkers Stratified by Age, Sex and PDF

Biomarker	Stratified covariate	Strata	*n*	*B*	*SE* Coeff	ß	*t*	*p*	Adj R^2 for model
Total leukocyte count, ×10^9/L	Age	1 (44.49-52.97 yr.)	7,749	-0.009	.001	-0.066	-6.204	<.001	.183
		2 (52.97-61.94 yr.)	7,752	-0.007	.001	-0.055	-5.124	<.001	.179
		3 (61.95-73.60 yr.)	7,749	-0.008	.001	-0.058	-5.214	<.001	.114
	Sex	Male	8,669	-0.006	.001	-0.045	-4.522	<.001	.172
		Female	14,581	-0.009	.001	-0.069	-8.940	<.001	.155
	PDF	1 (0.0-35.36 %)	7,750	-0.003	.003	-0.011	-1.077	.282	.165
		2 (35.36-45.07 %)	7,750	-0.010	.006	-0.018	-1.689	.091	.152
		3 (45.07-90.37 %)	7,750	-0.009	.002	-0.042	-3.927	<.001	.138
Neutrophil-to-lymphocyte ratio	Age	1 (44.49-52.97 yr.)	7,749	-0.003	.001	-0.046	-3.937	<.001	.011
		2 (52.97-61.94 yr.)	7,752	-0.003	.001	-0.046	-3.876	<.001	.030
		3 (61.95-73.60 yr.)	7,749	-0.003	.001	-0.042	-3.618	<.001	.038
	Sex	Male	8,669	-0.002	.001	-0.031	-2.886	.004	.036
		Female	14,581	-0.004	.001	-0.056	-6.743	<.001	.011
	PDF	1 (0.0-35.36 %)	7,750	-0.002	.002	-0.012	-1.023	.306	.018
		2 (35.36-45.07 %)	7,750	-0.007	.003	-0.027	-2.404	.016	.015
		3 (45.07-90.37 %)	7,750	-0.003	.001	-0.025	-2.225	.026	.010
Ln C-reactive protein	Age	1 (46.04-54.03 yr.)	1,398	-0.004	.002	-0.051	-1.926	.054	.126
		2 (54.03-61.10 yr.)	1,399	-0.005	.002	-0.060	-2.241	.025	.090
		3 (61.10-68.05 yr.)	1,399	-0.005	.002	-0.053	-2.036	.042	.102
	Sex	Male	1,652	-0.005	.002	-0.054	-2.269	.023	.119
		Female	2,544	-0.004	.002	-0.049	-2.590	.010	.126
	PDF	1 (7.39-36.26 %)	1,398	-0.014	.005	-0.071	-2.776	.006	.115
		2 (36.26-46.12 %)	1,399	-0.010	.009	-0.029	-1.151	.250	.120
		3 (46.14-87.84 %)	1,399	-0.003	.004	-0.019	-0.740	.460	.099

Note. Association between Paleolithic Diet Fraction (PDF) and inflammatory biomarkers stratified by age (tertiles), sex, and PDF (tertiles) in the study population and the C-reactive protein (CRP) subpopulation comprised of participants from the Malmö Diet and Cancer Study (MDCS) without previous coronary events, diabetes, stroke, or high-grade inflammation, and with no missing covariate data at baseline (1992-96) assessed through multivariable linear regression (adjusted for age, sex, physical activity level, body mass index, smoking status, education level, living alone, born in Sweden, season of dietary data collection, and dietary method version).
