# Supplementary material for: Association between Paleolithic diet fraction and systemic low-grade chronic inflammation in the Malmö diet and cancer study cohort
Source: Eur J Nutr. 2025 Nov 12;64(8):317. doi: 10.1007/s00394-025-03838-z (PMC12612027; doi:10.1007/s00394-025-03838-z)
Supplement: Supplementary file 2 — Supplementary file2 (PDF 80 KB) [file 394_2025_3838_MOESM2_ESM.pdf]

Table S2

Association Between Food Groups and Inflammatory Biomarkers

Food group	Biomarker	*n*	*B*	*SE* Coeff	ß	*t*	*p*	Adj R^2 for model
	Total leukocyte count (×10^9/L)	23,250	-0.001	.000	-0.039	-6.114	<.001	.156
Vegetables, g	Neutrophil-to-lymphocyte ratio	23,250	0.000	.000	-0.030	-4.404	<.001	.018
	Ln C-reactive protein	4,196	0.000	.000	-0.047	-3.131	.002	.113
	Total leukocyte count (×10^9/L)	23,250	0.000	.000	-0.019	-3.021	.003	.155
Fruits, g	Neutrophil-to-lymphocyte ratio	23,250	0.000	.000	-0.017	-2.455	.014	.017
	Ln C-reactive protein	4,196	0.000	.000	-0.037	-2.410	.016	.113
	Total leukocyte count (×10^9/L)	23,250	0.000	.000	0.023	3.484	<.001	.155
Potatoes, g	Neutrophil-to-lymphocyte ratio	23,250	0.000	.000	0.020	2.792	.005	.017
	Ln C-reactive protein	4,196	0.000	.000	-0.007	-0.469	.639	.111
	Total leukocyte count (×10^9/L)	23,250	0.000	.000	0.005	0.788	.431	.155
Eggs, g	Neutrophil-to-lymphocyte ratio	23,250	0.000	.000	-0.008	-1.264	.206	.017
	Ln C-reactive protein	4,196	0.000	.001	0.003	0.227	.821	.111
	Total leukocyte count (×10^9/L)	23,250	0.000	.000	0.011	1.633	.102	.155
Meat, g	Neutrophil-to-lymphocyte ratio	23,250	0.000	.000	-0.002	-0.251	.802	.017
	Ln C-reactive protein	4,196	0.001	.000	0.029	1.841	.066	.112
	Total leukocyte count (×10^9/L)	23,250	-0.001	.000	-0.019	-3.119	.002	.155
Fish, g	Neutrophil-to-lymphocyte ratio	23,250	-0.001	.000	-0.037	-5.530	<.001	.018
	Ln C-reactive protein	4,196	-0.001	.000	-0.034	-2.321	.020	.113
	Total leukocyte count (×10^9/L)	23,250	-0.010	.007	-0.009	-1.417	.156	.155
Olive and rapeseed oil, g	Neutrophil-to-lymphocyte ratio	23,250	-0.008	.004	-0.015	-2.362	.018	.017
	Ln C-reactive protein	4,196	0.006	.012	0.007	0.478	.632	.111
	Total leukocyte count (×10^9/L)	23,250	-0.004	.002	-0.013	-2.078	.038	.155
Nuts, g	Neutrophil-to-lymphocyte ratio	23,250	-0.002	.001	-0.010	-1.582	.114	.017
	Ln C-reactive protein	4,196	-0.005	.003	-0.025	-1.679	.093	.112
	Total leukocyte count (×10^9/L)	23,250	-0.001	.000	-0.065	-10.348	<.001	.158
Wine, g	Neutrophil-to-lymphocyte ratio	23,250	0.000	.000	-0.032	-4.763	<.001	.018
	Ln C-reactive protein	4,196	0.000	.000	-0.001	-0.060	.952	.111
	Total leukocyte count (×10^9/L)	23,250	0.001	.000	0.009	1.429	.153	.155
Legumes, g	Neutrophil-to-lymphocyte ratio	23,250	0.000	.000	0.007	1.004	.315	.017
	Ln C-reactive protein	4,196	0.000	.001	0.005	0.312	.755	.111
	Total leukocyte count (×10^9/L)	23,250	0.000	.000	0.016	2.617	.009	.155
Juice, g	Neutrophil-to-lymphocyte ratio	23,250	0.000	.000	0.011	1.681	.093	.017
	Ln C-reactive protein	4,196	0.000	.000	0.005	0.371	.710	.111
	Total leukocyte count (×10^9/L)	23,250	0.002	.000	0.032	5.009	<.001	.155
Meat products, g	Neutrophil-to-lymphocyte ratio	23,250	0.001	.000	0.026	3.712	<.001	.017
	Ln C-reactive protein	4,196	0.001	.000	0.022	1.399	.162	.112
	Total leukocyte count (×10^9/L)	23,250	0.000	.000	0.046	7.518	<.001	.157
Milk and milk products, g	Neutrophil-to-lymphocyte ratio	23,250	0.000	.000	0.019	2.972	.003	.017
	Ln C-reactive protein	4,196	0.000	.000	0.042	2.870	.004	.113
	Total leukocyte count (×10^9/L)	23,250	0.000	.000	0.037	6.058	<.001	.156
Sweet beverages, g	Neutrophil-to-lymphocyte ratio	23,250	0.000	.000	0.017	2.626	.009	.017
	Ln C-reactive protein	4,196	0.000	.000	0.018	1.195	.232	.112
	Total leukocyte count (×10^9/L)	23,250	0.000	.000	-0.004	-0.588	.557	.155
Cereals, g	Neutrophil-to-lymphocyte ratio	23,250	0.000	.000	0.011	1.502	.133	.017
	Ln C-reactive protein	4,196	0.000	.000	-0.029	-1.795	.073	.112
	Total leukocyte count (×10^9/L)	23,250	0.000	.000	-0.006	-1.019	.308	.155
Fats and oils, g	Neutrophil-to-lymphocyte ratio	23,250	0.001	.000	0.023	3.364	<.001	.017
	Ln C-reactive protein	4,196	0.000	.001	-0.001	-0.088	.930	.111
	Total leukocyte count (×10^9/L)	23,250	0.000	.000	0.012	1.981	.048	.155
Bakery sweets, g	Neutrophil-to-lymphocyte ratio	23,250	0.000	.000	0.021	3.175	.002	.017
	Ln C-reactive protein	4,196	0.000	.000	0.005	0.346	.730	.111
	Total leukocyte count (×10^9/L)	23,250	-0.001	.001	-0.016	-2.611	.009	.155
Jam, g	Neutrophil-to-lymphocyte ratio	23,250	0.001	.000	0.020	3.042	.002	.017
	Ln C-reactive protein	4,196	-0.001	.001	-0.028	-1.853	.064	.112
	Total leukocyte count (×10^9/L)	23,250	0.000	.000	0.009	1.433	.152	.155
Sauces and soups, g	Neutrophil-to-lymphocyte ratio	23,250	0.000	.000	0.006	0.940	.347	.017
	Ln C-reactive protein	4,196	0.000	.000	-0.005	-0.369	.712	.111
	Total leukocyte count (×10^9/L)	23,250	0.000	.000	-0.014	-2.132	.033	.155
Beer, g	Neutrophil-to-lymphocyte ratio	23,250	0.000	.000	0.001	0.139	.889	.017
	Ln C-reactive protein	4,196	0.000	.000	0.006	0.395	.693	.111
	Total leukocyte count (×10^9/L)	23,250	-0.002	.001	-0.018	-2.816	.005	.155
Spirits, g	Neutrophil-to-lymphocyte ratio	23,250	0.000	.000	-0.011	-1.586	.113	.017
	Ln C-reactive protein	4,196	0.000	.001	0.004	0.277	.782	.111
	Total leukocyte count (×10^9/L)	23,250	0.000	.001	-0.001	-0.218	.827	.155
Miscellaneous, g	Neutrophil-to-lymphocyte ratio	23,250	0.000	.001	0.004	0.670	.503	.017
	Ln C-reactive protein	4,196	0.004	.002	0.027	1.796	.073	.112
	Total leukocyte count (×10^9/L)	23,250	0.000	.000	-0.040	-6.265	<.001	.156
Paleolithic food groups, g	Neutrophil-to-lymphocyte ratio	23,250	0.000	.000	-0.032	-4.742	<.001	.018
	Ln C-reactive protein	4,196	0.000	.000	-0.041	-2.744	.006	.113
	Total leukocyte count (×10^9/L)	23,250	0.000	.000	0.048	7.315	<.001	.156
Non-Paleolithic food groups, g	Neutrophil-to-lymphocyte ratio	23,250	0.000	.000	0.034	4.802	<.001	.018
	Ln C-reactive protein	4,196	0.000	.000	0.037	2.335	.020	.113

Note. Association between food groups and inflammatory biomarkers in the study population and the C-reactive protein (CRP) subpopulation comprised of participants from the Malmö Diet and Cancer Study (MDCS) without previous coronary events, diabetes, stroke, or high-grade inflammation, and with no missing covariate data at baseline (1992-96) assessed through multivariate linear regression analysis (adjusted for age, sex, physical activity level, body mass index, smoking status, education level, living alone, bonr in Sweden, season of dietary data collection, and dietary method version).
